# COMPASSIONATE LOVE AND INTERSECTIONAL INEQUALITIES IN LATER-LIFE MENTAL HEALTH IN THE US

**DOI:** 10.1093/geroni/igae098.0138

**Published:** 2024-12-31

**Authors:** Tirth Bhatta, Jeffrey Albert, Stacy Torres, Eva Kahana, Nirmala Lekhak, Timothy Goler

**Affiliations:** University of Nevada Las Vegas, Las Vegas, Nevada, United States; Case Western Reserve University, Cleveland, Ohio, United States; University of California San Francisco, San Francisco, California, United States; Case Western Reserve University, Cleveland, Ohio, United States; University of Nevada Las Vegas, Las Vegas, Nevada, United States; Norfolk State University, Norfolk, Virginia, United States

## Abstract

Objectives: Despite the emerging evidence that compassionate love has a positive effect on mental health, its distribution across race, gender, and socioeconomic status and its subsequent role in mediating the effect of socioeconomic status on later life mental health across race and gender have yet to be examined. Methods: Based on our nationwide web-based survey of adults aged 50 years and older (n=1861), we conducted a mediation analysis to estimate the direct and indirect effects (via feeling love) of financial strain on depressive symptoms and anxiety across race and gender. Results: Our regression estimates indicate an average.09-point (p=.002) lower feeling of compassionate love among individuals experiencing increased financial strain. Women reported greater feeling of love (β=.22, p<.001) than men. Despite experiencing higher levels of financial strain, Black adults reported greater feelings of love (β=.42, p<.001) than white adults. Findings show an average.14-point (p<.001) and.21-point (p<.001) higher depressive symptoms and anxiety, respectively, among individuals experiencing increased financial strain. We found a statistically significant path-specific effect on both depressive symptoms (indirect effect=.018, p-value=.003) and anxiety (indirect effect=.196, p<.001) (from financial strain) through love. Statistically significant indirect effects through love on both depressive symptoms and anxiety were found for women and white adults, but not for men and Black adults. Discussion: Our study opens up a new avenue of research by investigating the role of compassionate love in transmitting the effects of financial strain on later-life mental health across race and gender.

